# The single mitochondrion of the kinetoplastid parasite *Crithidia fasciculata* is a dynamic network

**DOI:** 10.1371/journal.pone.0202711

**Published:** 2018-12-28

**Authors:** John DiMaio, Gordon Ruthel, Joshua J. Cannon, Madeline F. Malfara, Megan L. Povelones

**Affiliations:** 1 Sciences Division, Brandywine Campus, The Pennsylvania State University, Media, Pennsylvania, United States of America; 2 Department of Pathobiology, University of Pennsylvania School of Veterinary Medicine, Philadelphia, Pennsylvania, United States of America; Universidad Pablo de Olavide, SPAIN

## Abstract

Mitochondria are central organelles in cellular metabolism. Their structure is highly dynamic, allowing them to adapt to different energy requirements, to be partitioned during cell division, and to maintain functionality. Mitochondrial dynamics, including membrane fusion and fission reactions, are well studied in yeast and mammals but it is not known if these processes are conserved throughout eukaryotic evolution. Kinetoplastid parasites are some of the earliest-diverging eukaryotes to retain a mitochondrion. Each cell has only a single mitochondrial organelle, making them an interesting model for the role of dynamics in controlling mitochondrial architecture. We have investigated the mitochondrial division cycle in the kinetoplastid *Crithidia fasciculata*. The majority of mitochondrial biogenesis occurs during the G1 phase of the cell cycle, and the mitochondrion is divided symmetrically in a process coincident with cytokinesis. Live cell imaging revealed that the mitochondrion is highly dynamic, with frequent changes in the topology of the branched network. These remodeling reactions include tubule fission, fusion, and sliding, as well as new tubule formation. We hypothesize that the function of this dynamic remodeling is to homogenize mitochondrial contents and to facilitate rapid transport of mitochondria-encoded gene products from the area containing the mitochondrial nucleoid to other parts of the organelle.

## Introduction

Mitochondria are essential organelles for most eukaryotic cells. A typical cell contains a dynamic population of mitochondria whose architecture and distribution are maintained by protein-mediated mechanisms including fusion and fission events, mitochondrial movement, and positional tethering [1, 2]. The balance between membrane fusion and fission reactions, collectively called mitochondrial dynamics, is of particular importance as it contributes to several essential functions [3]. First, mitochondrial dynamics helps to establish mitochondrial morphology, which is closely linked to function [4–6]. Elaborate mitochondrial networks are associated with high rates of aerobic respiration, while more simplified mitochondria occur in cells with low respiratory activity [7]. Mitochondrial dynamics are also coordinated with the cell cycle in order to ensure the even distribution of mitochondria into daughter cells [8–10]. Fusion homogenizes proteins and other macromolecules, which is important for overall mitochondrial health and network function [11–14]. In contrast, fission is increased during mitosis to allow the stochastic partitioning of organelles into daughter cells. Finally, mitochondrial dynamics allows for organelle quality control [15, 16] by rescuing [17–19] or disposing [3, 20] of mitochondrial fragments produced by depolarization of the membrane potential.

Mitochondrial fission and fusion are mediated by distinct members of the dynamin GTPase superfamily, and inhibition of these activities produces distinct morphological phenotypes. A lack of fission produces a more highly-fused, interconnected mitochondrial network, while interrupting fusion results in small, fragmented mitochondria [21–23]. Many other proteins have been implicated in mitochondrial dynamics [1, 6, 21, 24]. These studies have used model systems such as *Saccharomyces cerevisiae*, *Drosophila melanogaster*, and mammalian cells [25]. Much less is known about how and if mitochondrial dynamics occurs in other eukaryotic organisms. Kinetoplastid parasites are unusual among eukaryotes in that each cell contains a single mitochondrion. Thus, it is essential that mitochondrial biogenesis and division are coordinated with the cell cycle. This is also true for other organisms with single mitochondria, such as *Toxoplasma gondii* [26]. For example, in *Trypanosoma brucei*, mitochondrial fission mediates the final division event that splits one mitochondrion into two immediately prior to cytokinesis. This process is mediated by a dynamin-like protein called TbDLP. In other eukaryotes, dynamins that function in mitochondrial dynamics differ from the classical dynamins that mediate vesicle fission during endocytosis. Interestingly, *T*. *brucei* and other kinetoplastids lack classical dynamins [27, 28]. In fact, most kinetoplastids encode a single DLP, suggesting that a single enzyme can function in both mitochondrial fission and endocytosis, as has been demonstrated for bloodstream form *T*. *brucei* [29, 30]. Furthermore, kinetoplastid genomes lack identifiable orthologs for most other mitochondrial dynamics proteins, leading some to conclude that conventional fission and fusion outside of organelle division do not occur in these organisms [30, 31]. However, mitochondrial dynamics has been demonstrated in plants despite a lack of orthologs for proteins expected to mediate these processes [32].

We are interested in the inherent properties of mitochondrial networks and in exploring the unique challenges faced by eukaryotic organisms with a single mitochondrion and mitochondrial nucleoid. For this, we decided to work with the model kinetoplastid *Crithidia fasciculata*, which is a monoxenous parasite of mosquitoes. These parasites live on flowers, fruit, or in aquatic environments before being taken up by a mosquito [33]. *C*. *fasciculata* presents several practical advantages for investigating kinetoplastid cell biology. It can be grown in large quantities, it is genetically tractable, and its cell cycle can be easily synchronized. They have two developmental forms, a swimming nectomonad and a non-motile haptomonad, both of which can be generated in culture [34]. The haptomonad stage is particularly well-suited for live-cell imaging.

Here we describe the mitochondrial division cycle in *C*. *fasciculata*. We found that mitochondrial biogenesis is coordinated with the cell cycle and occurs mainly in G1, while mitochondrial division occurs shortly before cytokinesis. We observed through examination of live cells that the mitochondrion is dynamic and is constantly remodeled through both fission and fusion events. Furthermore, we have visualized the complex process of mitochondrial DNA division in these cells. Collectively, these observations provide a new perspective on the kinetoplastid mitochondrion and reveal intriguing avenues of investigation. The study of mitochondrial dynamics in early-diverging eukaryotes provides an important evolutionary perspective on both the function and mechanisms of these processes and may have broad relevance for other eukaryotic systems.

## Materials and methods

### Parasite growth

*C*. *fasciculata* strain CfC1 was grown in brain heart infusion (BHI) medium supplemented with 20 μg/ml bovine hemin (Sigma) at 27°C. Cells grown on a rocker were passaged as nectomonads every 2–3 days to maintain a density between 10^5^ and 10^8^ cells/ml. Cell densities were determined by mixing a small sample of cells with an equal volume of 3% formalin, followed by staining with crystal violet before loading samples on a hemacytometer for counting. To generate adherent haptomonads for imaging, 2 ml of culture at a density of 1 x 10^7^ cells/ml was seeded in a 35 mm poly-L-lysine-coated MatTek dish. The cells were allowed to adhere for 2 hours without rocking. Following 3 washes with BHI, fresh media was added, and dishes were incubated without rocking for 24 hours prior to imaging. To obtain clonal *C*. *fasciculata* cell lines, serial dilutions of culture were plated on BHI plates made with 0.65% agarose and supplemented with hygromycin (Amresco) [35]. After 4–5 days of growth, parasite colonies were transferred with a pipet tip to a standard liquid culture containing hygromycin.

### Plasmids and cell lines

To obtain cells expressing mitochondrial GFP, the mitoGFP gene was amplified from the pLew100mitoGFP plasmid (a gift from Stephen Hajduk, [36]) using primers 5´- TATGGTACCtcgtcccgggctgcacg-3´ and 5´-TATCTCGAGcgcacctccctgctgtgcc-3´ and cloned into pNUSHcH (a gift from Emmanuel Tetaud, [37]) with KpnI and XhoI. The resulting pNUSmitoGFPcH was introduced into *C*. *fasciculata* CfC1 cells by electroporation (modified from [38]). Briefly, 5 x 10^7^ cells were centrifuged at 1000 rcf for 5 min, washed once with 1 ml electroporation buffer (21 mM HEPES pH 7.4, 137 mM NaCl, 5 mM KCl, 0.7 mM Na_2_PO_4_, 6 mM glucose), and resuspended in 1 ml electroporation buffer. 2.5 x 10^7^ cells were electroporated in a 4 mm gap cuvette containing 40 μg of purified plasmid using a BTX ECM-600 electroporator with a single pulse (475 V, 800 μF, 13 Ω). Cells were allowed to recover for 18–24 hours in BHI supplemented with 10% FBS. Following recovery, hygromycin was added to a final concentration of 80 μg/ml. Transfected cells were passaged 7–12 days later, at which point the hygromycin concentration was increased to 200 μg/ml to select for cells with stronger GFP expression. This cell line was cloned by limiting dilution onto BHI agarose plates to create the cell line CfmitoGFP2.6. A modification of the pNUSmitoGFPcH plasmid containing a ribosomal DNA promoter upstream of the mitoGFP gene was created by amplifying the rDNA promoter from *C*. *fasciculata* genomic DNA using primers 5´-TATTATAAGCTTctgggttatagggggatgct-3´ and 5´-TATTATAAGCTTatcaatcaagtgccgactcc [39, 40] -3´. This fragment was introduced into the HindIII site of pNUSmitoGFPcH to create pNUSrpMitoGFPcH. This plasmid was introduced into the CfC1 line using nucleofection with a Lonza IIb Nucleofector device, the Lonza Human T-cell kit, and program X-001 according to the manufacturer’s protocol. Cells were allowed to recover as described above. The resulting CfrpMitoGFP line (non-clonal) showed comparable fluorescent intensity and pattern as CfmitoGFP2.6, therefore both cell lines were used interchangeably in this study.

### Western blot analysis

To screen clones of CfmitoGFP for maximal expression of the fluorescent protein, protein lysates were created from populations and clones of both the parental CfC1 line and the mitoGFP line. 2 x 10^7^ cells per sample were centrifuged, washed once with PBS, and resuspended in 200 μl of hot Laemmli SDS-PAGE sample buffer. Samples were boiled for 5 min and cleared by centrifugation at 21,000 rcf. 1 x 10^6^ cell equivalents per lane were run on a 12% SDS-PAGE gel. An identical gel was run and Coomassie-stained to confirm equal loading. Resolved proteins were transferred onto PVDF, which was blocked with PBS containing 5% non-fat dry milk and 1% Tween-20 (PBST) and probed with mouse-anti-GFP (Roche) at a dilution of 1:250 in PBST containing 1% milk. The blot was probed with anti-mouse-HRP (Jackson ImmunoResearch) at a dilution of 1:5000, incubated with ECL (BioRad), and imaged with a G:Box gel imager.

### Cell fixation and imaging

To fix cells for fluorescent microscopy, 1 x 10^7^ cells for each sample were centrifuged at 1000 rcf for 5 min and resuspended in 1 ml of BHI. 36% formaldehyde (Sigma-Aldrich) was added to a final concentration of 4% and samples were incubated at room-temperature for 5 min. Fixed cells were centrifuged again and washed twice with 1 ml of PBS. Cells were resuspended in PBS at a density of 1 x 10^7^ cells/ml. 200 μl of this mixture was added to a charged slide (Thermo), which was incubated in a humidified chamber for 10–20 min. Slides were washed once briefly and again for 5 min in PBS in Coplin jars. To permeabilize cells, 200 μl of PBS containing 0.1% Triton X-100 was added to the slides and incubated for 5 min. Slides were then washed as before and stained with 0.2 μg/ml DAPI in PBS for 5 min. Slides were washed again and mounted in 90% glycerol in PBS. Wide-field fluorescent imaging was performed on a Zeiss Axioscope.A1 upright LED fluorescence microscope equipped with a Zeiss AxioCam ICm1 camera. Confocal imaging of fixed cells was performed on a Leica SP5-II confocal microscope. Length and width of cells was measured in ImageJ. To measure mitochondrial area, maximum projections of confocal Z-stacks were generated in ImageJ. After applying a fluorescence threshold, the area was calculated. Nodes were counted manually in ImageJ max projections. Statistical analyses were performed using GraphPad Prism. Data from individual samples was first analyzed for normality using the D’Agostino & Pearson normality test. If all samples being analyzed showed a normal distribution, an ANOVA multivariable analysis or an unpaired t-test was used to test for significance. If at least one sample did not show a normal distribution, a Mann-Whitney test was used.

### Synchronization

Synchronization was performed as in [41]. Briefly, 2.5 mM (final) hydroxyurea (HU, Sigma) was added to a 20 ml culture at a density of 1 x 10^7^ cells/ml. After six hours of treatment, cells were centrifuged, washed once with 20 ml BHI without HU and then resuspended in fresh BHI. A portion of culture was immediately removed and processed for fixed cell microscopy as described above (0 hour). Additional samples were removed and processed every 30 min until 150 min. A sample was also prepared from an asynchronous culture at comparable density. For each sample, two slides were made. One set of slides were analyzed by wide-field fluorescent microscopy to assess the efficiency of the synchronization procedure, while the other set was analyzed by confocal microscopy. For fluorescence microscopy-based quantitation the mean and standard error of three independent replicates is shown except for times 0 and 0.5 h post-release which were examined in two replicates.

### Live cell imaging

To immobilize swimming nectomonads for live cell imaging two methods were used. First, an agarose pad was prepared by adding 100 μL of BHI containing 1% low melting point agarose (IBI Scientific). This solution was boiled, equilibrated to 65°C, then added to a glass slide. Another slide was placed on top. After 10 min the top slide was gently removed and 20 μl of a mid-log phase culture (~3 x 10^7^ cells/ml) was added to the center of the agarose pad. A coverslip was added and sealed with VaLaP. In the second method, a BHI-agarose mixture was prepared as before, equilibrated to 65°C, then mixed 1:1 with a culture of swimming cells. 20 μl of this mixture was added to a slide, cover-slipped, and sealed with VaLaP. Both methods were reasonably effective at immobilizing living *C*. *fasciculata* parasites. Cells with motile flagella (an indication of cell viability) but an immotile cell body were selected for imaging. For live-cell imaging of adherent haptomonads, cultures were prepared as described above. Cells were imaged with either a Yokagawa CSU X-1 spinning disk confocal head configured on an inverted Leica DMI4000 microscope with a Hamamatsu EM512 camera or a Leica SP5 II laser confocal microscope on a DMI6000 stand as indicated. On the spinning disk confocal Z-stacks were taken every 2 min for up to 60 min without significant photobleaching or phototoxicity. On the laser scanning confocal Z-stacks were taken every 12–30 seconds for up to 40 min and a resonant scanner was used for rapid imaging. Both confocal systems were outfitted with environmental controls which were set to 27°C during imaging. Some time-lapse confocal images were processed with Huygens deconvolution software (Scientific Volume Imaging).

## Results

### Mitochondrial GFP is a marker for the *C*. *fasciculata* mitochondrion

**Fig 1:**
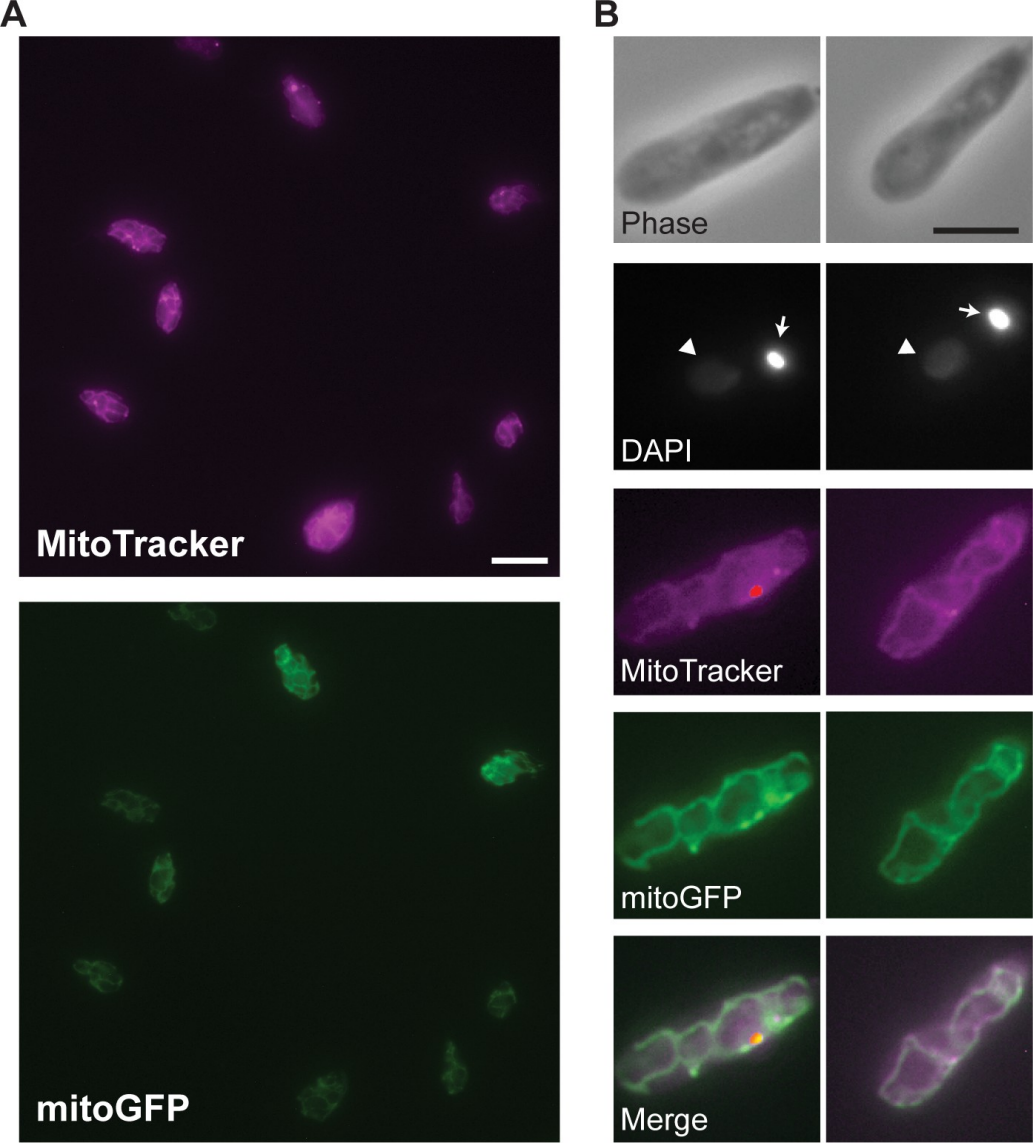
mitoGFP is a mitochondrial marker in *C*. *fasciculata*. A) Mitochondrial staining by the potential-dependent dye MitoTracker Red (MitoTracker, top panel) and by mitochondrial-targeted GFP (mitoGFP, lower panel). mitoGFP expression is driven by an ectopic plasmid causing heterogeneous fluorescence among cells. Scale bar is 10 μm. B) Higher magnification images of two representative cells show colocalization between MitoTracker and mitoGFP signals. The phase contrast image shows the cell boundaries. Nuclear DNA (arrowhead) and mitochondrial DNA (kDNA, arrow) are stained with DAPI. In merged images MitoTracker is shown in magenta and mitoGFP in green. Scale bar is 5 μm.

To image the *C*. *fasciculata* mitochondrion, we obtained a mitochondrial GFP (mitoGFP) construct in which a 14-amino acid mitochondrial targeting sequence from *T*. *brucei* is fused to GFP [36], targeting the protein to the mitochondrial matrix. The activity-based dye MitoTracker Red CMXRos also labels the mitochondrion, but in *C*. *fasciculata* tends to accumulate in one or two bright puncta that saturate before the rest of the organelle shows robust signal. We therefore created a drug-selectable mitoGFP expression construct for *C*. *fasciculata* [37]. After introducing this construct into wild-type cells, we observed a fluorescence pattern consistent with kinetoplastid mitochondria and which colocalized with MitoTracker Red (Fig 1). Expression of mitoGFP is heterogeneous within the cell population, as one would expect for an episomal construct (Fig 1A, [37]). By screening clones of this cell line, we were able to select a clone with a higher overall signal, presumably because it maintains more copies of the expression construct (S1 Fig). In parallel studies we modified our expression construct by introducing an rDNA promoter upstream of the mitoGFP gene. This resulted in a strong, yet still heterogeneous, signal that also colocalized with MitoTracker Red. Both cell lines are used in this study as they showed an identical pattern and were of comparable brightness. We saw no association between the strength of the mitoGFP signal and the stage of the cell cycle.

### Mitochondrial biogenesis during the *C*. *fasciculata* cell cycle

**Fig 2:**
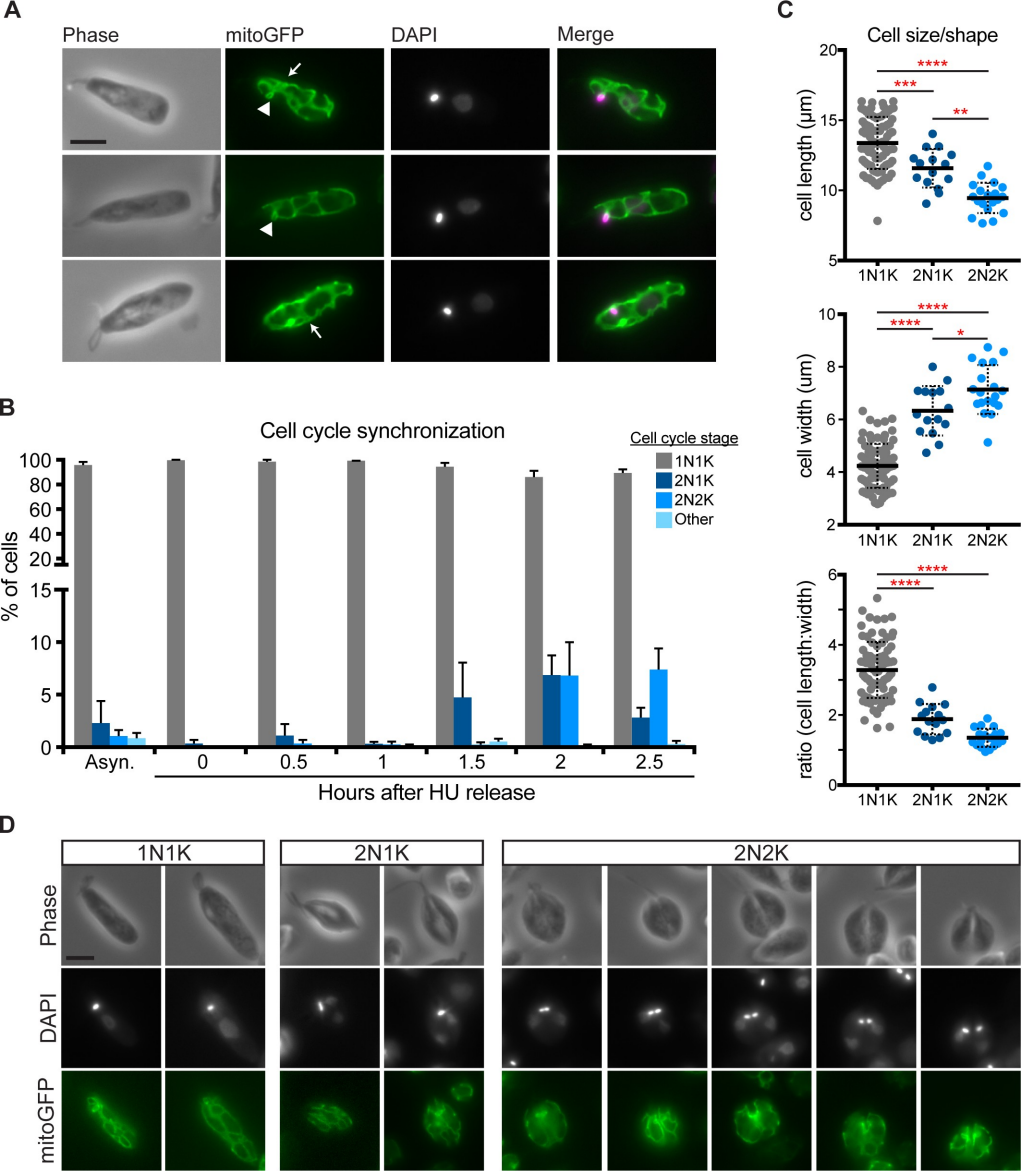
Mitochondrial shape during the cell cycle of *C*. *fasciculata*. A) Typical mitochondrial morphology in 1N1K (G1 phase) cells as shown by mitoGFP. Arrowheads show a small mitochondrial loop that indicates the presence of the kDNA disk. Arrows point to areas containing thickened mitochondrial tubules. DAPI signal is shown in magenta in merged images. Scale bar is 5 μm. B) Relative numbers of cells in different phases of the cell cycle in asynchronous cultures and in hydroxyurea (HU)-synchronized cultures at various times after removal of HU. This procedure allows for enrichment of mitotic cell cycle phases and demonstrates cell cycle progression from 1N1K to 2N1K, 2N2K, and back to 1N1K. N, nucleus. K, kDNA. C) Synchronized cells (n = 119) in different phases of the cell cycle according to DAPI staining were measured for overall cell length and width using phase contrast images. For length, cells were measured along their longest axis from the base of the flagellum to the posterior end. Cell width was measured at the widest point of the cell. P-values indicated *<0.05, **<0.005, *** = 0.001, and ****<0.0001 by ANOVA. D) Examples of typical mitochondrial shapes found in 1N1K, 2N1K, and 2N2K cells. Note that the nuclei are not symmetrically positioned with respect to the cell body in 2N1K cells. The last three columns show cells undergoing cytokinesis. Scale bar is 5 μm.

We first examined the overall shape of the mitochondrion in logarithmically-growing *C*. *fasciculata* cells. Consistent with what has been reported for other kinetoplastid parasites, we found that the mitochondrion takes the shape of a branched tubular network that extends throughout the cell (Fig 2A). In many cells, there was a clear space with fewer mitochondrial tubules in the part of the cell that contains the nucleus. We also noted that many cells contained areas of thickened mitochondrial tubules, although by widefield epifluorescence microscopy we could not rule out that these regions might be out of focus. This variability in mitochondrial diameter did not appear to associate with any particular phase of the cell cycle.

Because kinetoplastids, such as *C*. *fasciculata*, have only a single mitochondrion, mitochondrial biogenesis and division must be timed with the cell cycle. In addition, each mitochondrion contains a single mitochondrial nucleoid, called the kinetoplast DNA (kDNA), which consists of thousands of catenated DNA circles. It is well-known that replication and division of the kDNA also occurs within a particular cell cycle stage [42–45]. To describe the mitochondrial duplication and division cycle of *C*. *fasciculata*, we first examined asynchronous cultures of mitoGFP-expressing cells. Staining with DAPI and counting the number of nuclei and kDNA in each cell allows for a rough approximation of cell cycle stage. In *Crithidia*, as in *Leishmania* but unlike in *T*. *brucei*, the nucleus (N) divides first followed by the kDNA (K) [44]. Therefore, cells progress from 1N1K to 2N1K and 2N2K before undergoing cytokinesis and returning to 1N1K (Fig 2B). In asynchronous culture, more than 90% of the cell population is 1N1K, indicating that the stages following mitosis and kDNA division are brief (Fig 2B). To enrich for these stages, we synchronized our cultures using hydroxyurea (HU) [41, 43]. This nucleoside analog causes cells to arrest and accumulate in G1 (1N1K). Removal of the drug releases the block, allowing cells to progress through the cell cycle. Using our synchronization enrichment protocol, we were able to observe ~11% 2N1K cells at 1.5 h post-release and ~12% 2N2K cells at 2 h post-release (Fig 2B). This is compared to approximately 1% in asynchronous cultures.

When we analyzed mitoGFP cells by fluorescence microscopy, 2N1K and 2N2K cells appeared wider and shorter than 1N1K cells. To quantitate this, we measured the length and width of cells in different phases of the cell cycle in our partially synchronized cultures. Indeed, as cells progressed from 1N1K to 2N2K their average length decreased while their width increased (Fig 2C). This resulted in a morphologically symmetrical cell that can be divided almost exactly in half during cytokinesis, followed by cell elongation in G1. However, when we examined the pattern of DAPI staining in 2N1K cells we observed that the positioning of the nucleus was not perfectly symmetrical (Fig 2D). This asymmetry resolves as cells progress to the 2N2K stage, when the kDNA is positioned symmetrically over the cell division plane.

The cell cleavage furrow begins, as in other kinetoplastids [46], at the anterior end of the cell between the two flagella and moves posteriorly until the cells separate. During this process, the mitochondrion remains branched and appears to be equally partitioned between daughter cells (Fig 2D). Some mitochondrial tubules are found close to the cell periphery. In 2N2K cells, these tubules often formed a “V” shape, where the point of the “V” coincided with the progressing cytokinesis furrow (Fig 2D).

### Distinct mitochondrial morphologies in replicating *C*. *fasciculata*

To perform a more detailed analysis of mitochondrial morphology during the cell cycle, and to minimize the effects of out-of-focus light, we used confocal microscopy and deconvolution to image synchronized mitoGFP cells. We took confocal z-stacks through 83 1N1K cells, 15 2N1K cells, and 16 2N2K cells from two separate experiments. Consistent with our previous results, we observed that the majority of mitochondrial networks (~81%) were mixtures of thin tubules and thicker, flatter tubules, while the remaining cells contained mitochondrial tubules of fairly uniform diameter. We saw no clear association between thickened mitochondrial tubules and any particular region of the cell, although all 2N2K cells contained thicker tubules in the middle/midline region (S2 Fig).

**Fig 3:**
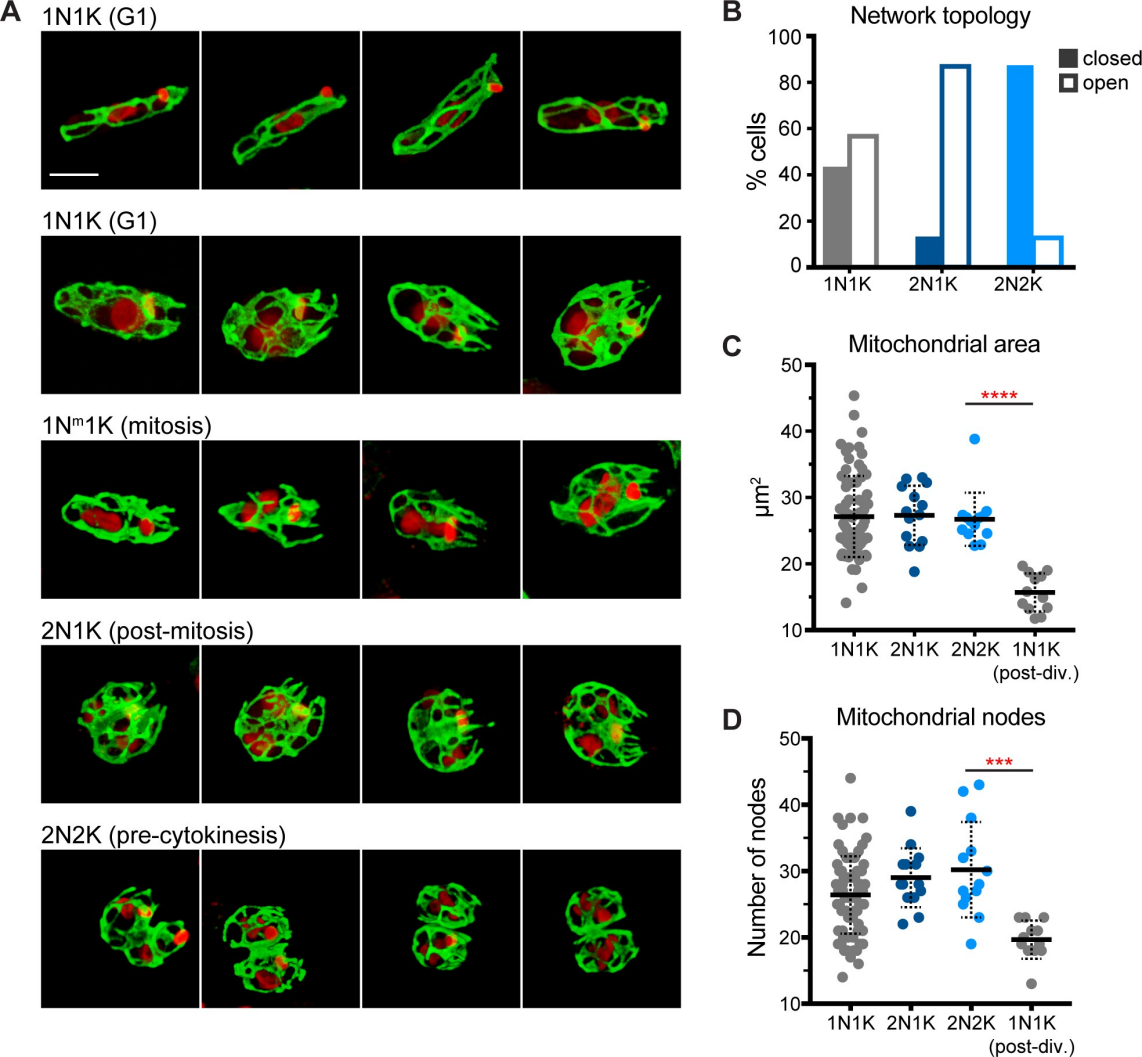
Confocal analysis of mitochondrial shape during the cell cycle of *C*. *fasciculata*. A) Examples of maximum projections of fixed, synchronized *C*. *fasciculata* mitoGFP-expressing cells as imaged by confocal microscopy. Mitochondria are shown in green and DAPI in red. Cell cycle stages are shown above each row. The first row shows all “closed” networks. Rows 2–4 show networks that are “open” at the anterior end (containing tubule end points rather than loops). Each image is oriented such that the anterior of the cell is on the right. Scale bar is 5 μm. B) Quantitation of the percent of cells (n = 114) in each cell cycle stage that have closed or open mitochondrial networks at their anterior end. C) Mitochondrial area in z-stack maximum projections as a function of cell cycle stage (n = 115). **** indicates P-value <0.0001 by Mann-Whitney test. D) The numbers of mitochondrial nodes, or branch points, were counted manually in maximum projections (n = 115) *** indicates P-value of 0.0002 by unpaired t-test with Welch’s correction.

This analysis also revealed some cell cycle stage-specific mitochondrial morphologies (Fig 3A and 3B). In 43% of G1 phase cells (1N1K), the mitochondrion is a network of closed loops. In the remaining cells, there were free mitochondrial ends extending towards the anterior end of the cell. These open networks were commonly found in wider cells with enlarged or mitotic nuclei. Consistent with this, in 2N1K cells there were a greater number of cells with open mitochondrial networks (87% in 2N1K compared to 57% in 1N1K). Closed networks predominate again during the 2N2K stage, with only 13% of 2N2K cells having open mitochondrial networks.

To examine mitochondrial biogenesis and topology during the cell cycle, we analyzed maximum projections of each z-stack to quantitate mitochondrial area and number of network branch points (Fig 3C and 3D). A branch point was defined as a position at which two or more mitochondrial tubules diverged. Interestingly, we found a wide range of mitochondrial areas in 1N1K cells compared to more narrow ranges for 2N1K and 2N2K cells. The mean mitochondrial area in each of these categories is approximately the same. When we separated 1N1K cells that had clearly just divided or were minimally connected to another 1N1K cell by a narrow cytoplasmic bridge and treated them as a separate category, we found that their mean mitochondrial area was approximately half that of the other cell types. This implies that the mitochondrion is divided in half during cell division, and then grows to its full size during G1. By the 2N1K stage, the mitochondrion has returned to its normal size, which is similar in all 2N1K cells analyzed. Similar to mitochondrial area, the number of mitochondrial branch points remained relatively constant except in recently divided cells, when it was halved.

### The *C*. *fasciculata* mitochondrion is a dynamic network

**Fig 4:**
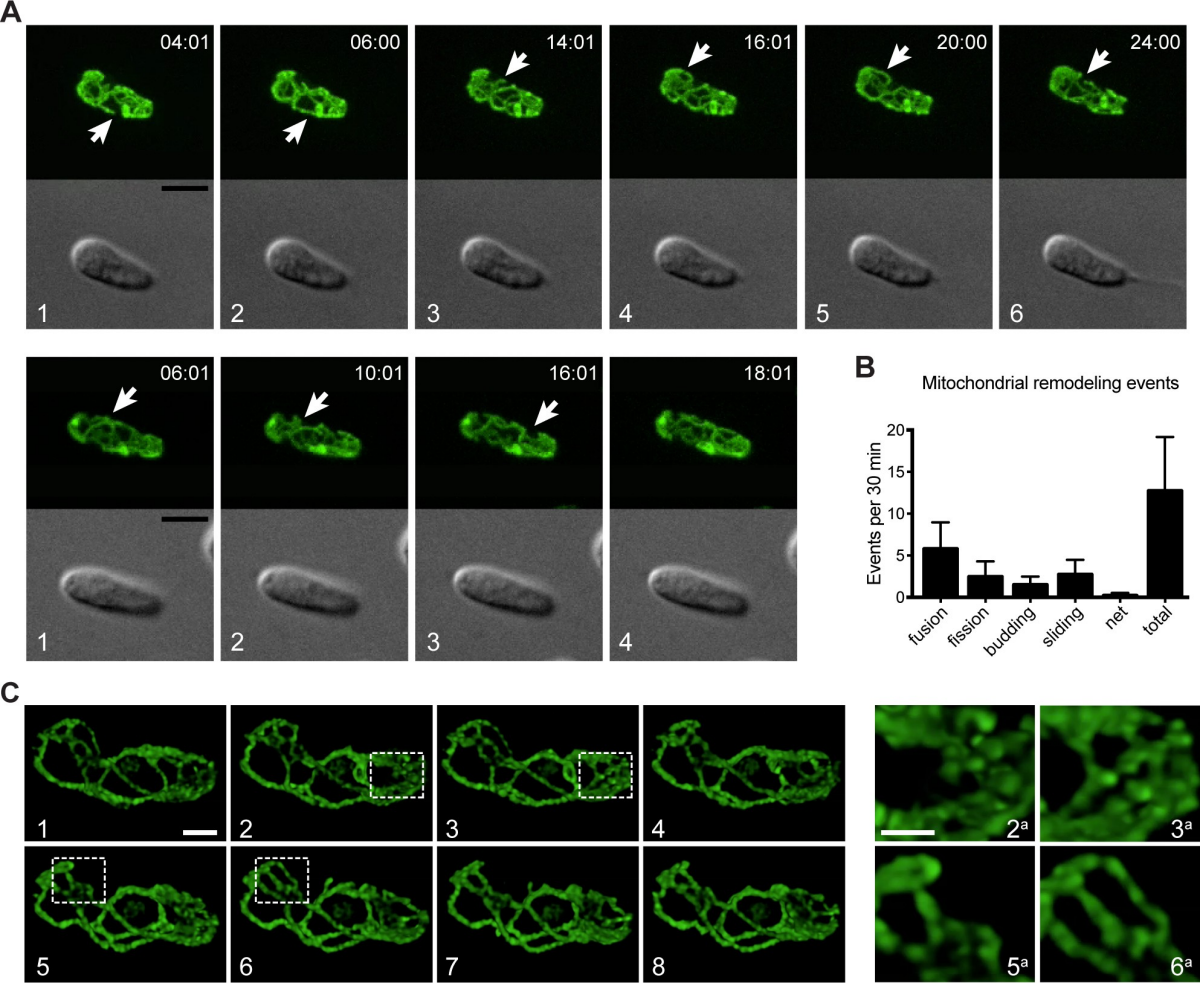
Mitochondrial dynamics in *C*. *fasciculata*. A) Frames from live-cell imaging of two cells by spinning-disk confocal microscopy. Cells are expressing mitoGFP. Mitochondrial remodeling reactions are indicated by arrows. Times are given as h:min:s. White numbers indicate frame sequence. Not all frames in the time-course are shown (see S1 and S2 Movies) Scale bar is 5 μm. B) Quantitation of mitochondrial remodeling events in spinning disk confocal experiments. Z-stacks of 17 cells were generated every 2 min for an average of 49 min. The resulting movies were then analyzed for different types of mitochondrial remodeling events. The frequency of each event per minute was calculated then multiplied by 30 to give number of events/30 min time period. The bar labeled total represents the sum of all the different types of events shown by the other bars. C) Live-cell imaging of mitochondrial remodeling with a laser-scanning confocal microscope and deconvolution. White numbers indicate different frames. Boxes indicate areas in which remodeling occurs between frames. These areas are shown enlarged (with corresponding numbers) in the right panels. Scale bars are 2 μm.

To address whether mitochondrial biogenesis in G1 phase cells requires events resembling mitochondrial dynamics, we imaged mitochondria in live *C*. *fasciculata*. For swimming nectomonads, we immobilized asynchronous mitoGFP parasites and imaged them on a confocal microscope (Fig 4). We observed that mitochondrial networks are dynamic even in G1 phase cells (those with a single flagellum, Fig 4A and S1 and S2 Movies). The topology of the network was constantly changing as tubules divided, fused, and emerged from existing tubules. Branch points were also observed to migrate along tubules. In some instances, complex net-like structures resembling fenestrated sheets of mitochondrion would appear transiently before resolving into tubules and loops. The position of the kDNA within the mitochondrial network was usually visible as a dark area surrounded by a small loop of fluorescence signal (Fig 4A). This has also been observed in *T*. *brucei* and was named the “kDNA pocket” [47]. The kDNA serves as a landmark, allowing us to ensure that dynamic events are not due to the cell rolling or shifting on the slide. We manually scored remodeling events in 17 cells to determine their frequency (Fig 4B). Though some cells were assayed for longer, we calculated the frequency of events during a 30-minute interval because photobleaching was negligible over this period. In total, the average length of time between network remodeling events of any category was 3.1 minutes, or 12.7 events per 30 minutes of observation. We found that tubule fusions were the most common event observed, with an average of 5.8 events in a 30-minute period. These fusion events included both side-to-side tubule fusions and end-to-side fusions. Over this same time period, fission, or division of a tubule, was observed an average of 2.5 times, while budding of a new tubule from an existing one was observed 1.5 times. Branch point sliding occurred with a greater frequency, with 2.8 events per 30 minutes, while the rarest event was the appearance of a fenestrated sheet, which was only observed in 6 of the 17 cells imaged and in those only once per observation period.

We also visualized these dynamic events using laser scanning confocal microscopy, enabling the use of deconvolution to view mitochondrial network remodeling in more detail (Fig 4C). This analysis revealed that some regions of the network were more dynamic than others, with rapid remodeling (mainly fusions, fissions, and branch point migration) occurring at the anterior and posterior ends of the cell, and less frequent remodeling in the center of the cell, where such events might be physically restricted by the presence of the nucleus.

### Mitochondrial dynamics in adherent, non-motile *C*. *fasciculata*

**Fig 5:**
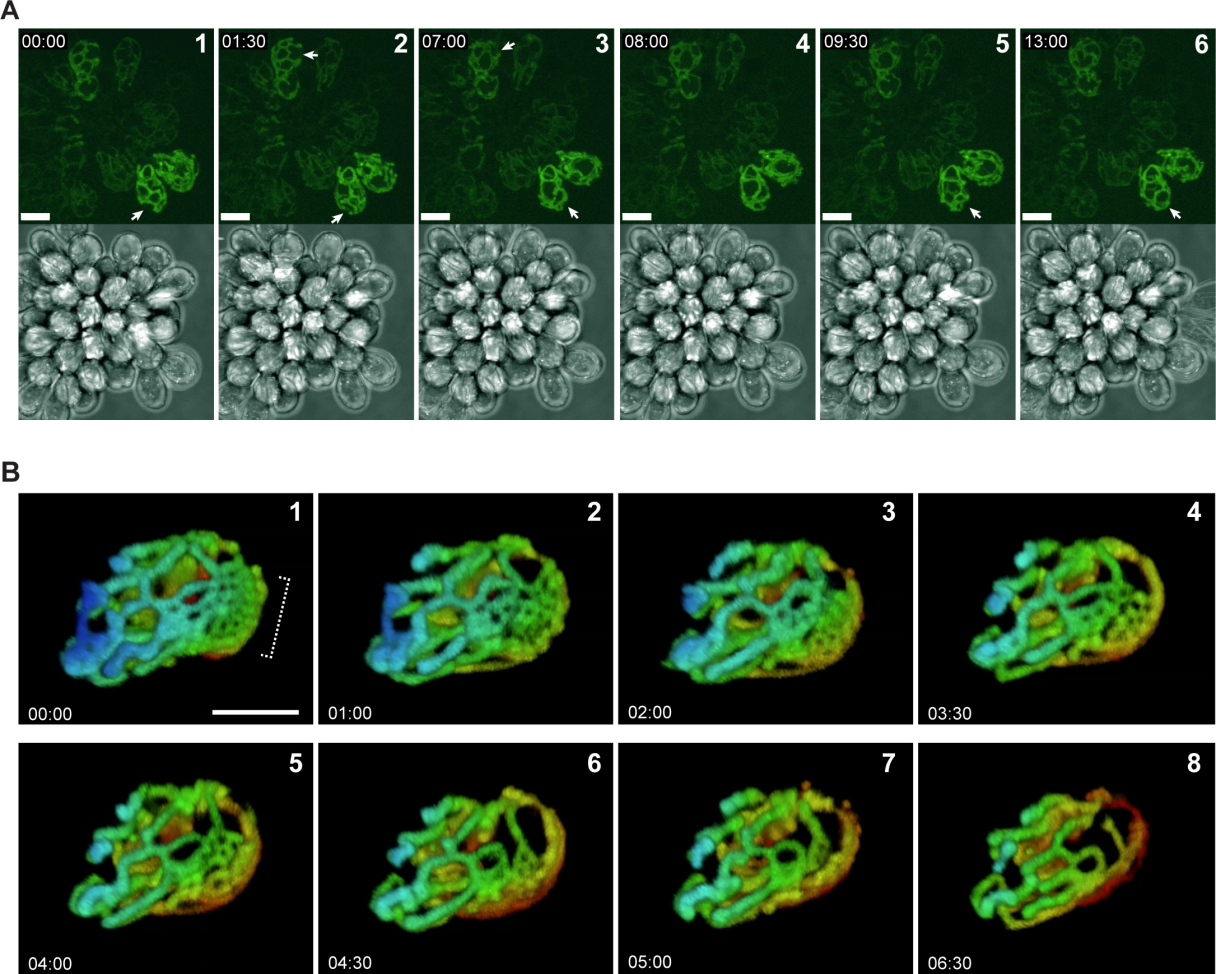
Adherent *C*. *fasciculata* haptomonads also show rapid mitochondrial remodeling. A) Max projections of a typical rosette of *C*. *fasciculata* imaged using a laser scanning confocal microscope. Mitochondria in live cells were visualized using mitoGFP. Mitochondrial remodeling reactions are indicated by arrows. Times are given as mm:ss. White numbers indicate the sequence of frames. Note that not every frame is shown (see S3 Movie). Scale bar is 10 μm. B) Time course of mitochondrial remodeling in a single adherent cell. Each frame is a max projection that has been color-coded by depth. White numbers indicate the sequence of frames. Note that not every frame is shown (see S4 Movie). Bracket indicates the formation of a net-like structure that then is rapidly remodeled to tubules during the course of our observation. Scale bar is 5 μm.

An advantage of working with *C*. *fasciculata* is that cultured cells will readily differentiate into a naturally adherent, non-motile stage [34]. If culture flasks are agitated with gentle shaking, the majority of cells will remain in the swimming or nectomonad form, which is highly motile with a full-length flagellum. In stationary cultures, a fraction of cells will adhere to the tissue culture plastic via their flagellum and differentiate into the non-motile haptomonad form. Haptomonds can grow and divide while adhered to a plastic or glass surface. The flagellum in haptomonads is dramatically shorter, and usually does not emerge from the cellular invagination called the flagellar pocket. We observed live mitoGFP-expressing haptomonads by confocal microscopy and found that their mitochondrion was a branched network similar to that of nectomonads. In addition, remodeling events consistent with mitochondrial dynamics were readily apparent (Fig 5A and S3 and S4 Movies). In particular, the area of the mitochondrion containing the kDNA was very kinetic, appearing as a loop of mitochondrial matrix at the end of a mitochondrial tubule stalk. The movement of the kDNA disk may be due to motility of the shortened haptomonad flagellum, or through another, unknown mechanism. We observed similar classes of remodeling events as were seen in nectomonads, including the transient appearance of fenestrated sheets of mitochondrion, which quickly resolved into tubules (Fig 5B and S4 Movie).

### The coordination of kDNA division, mitochondrial division, and cytokinesis

**Fig 6:**
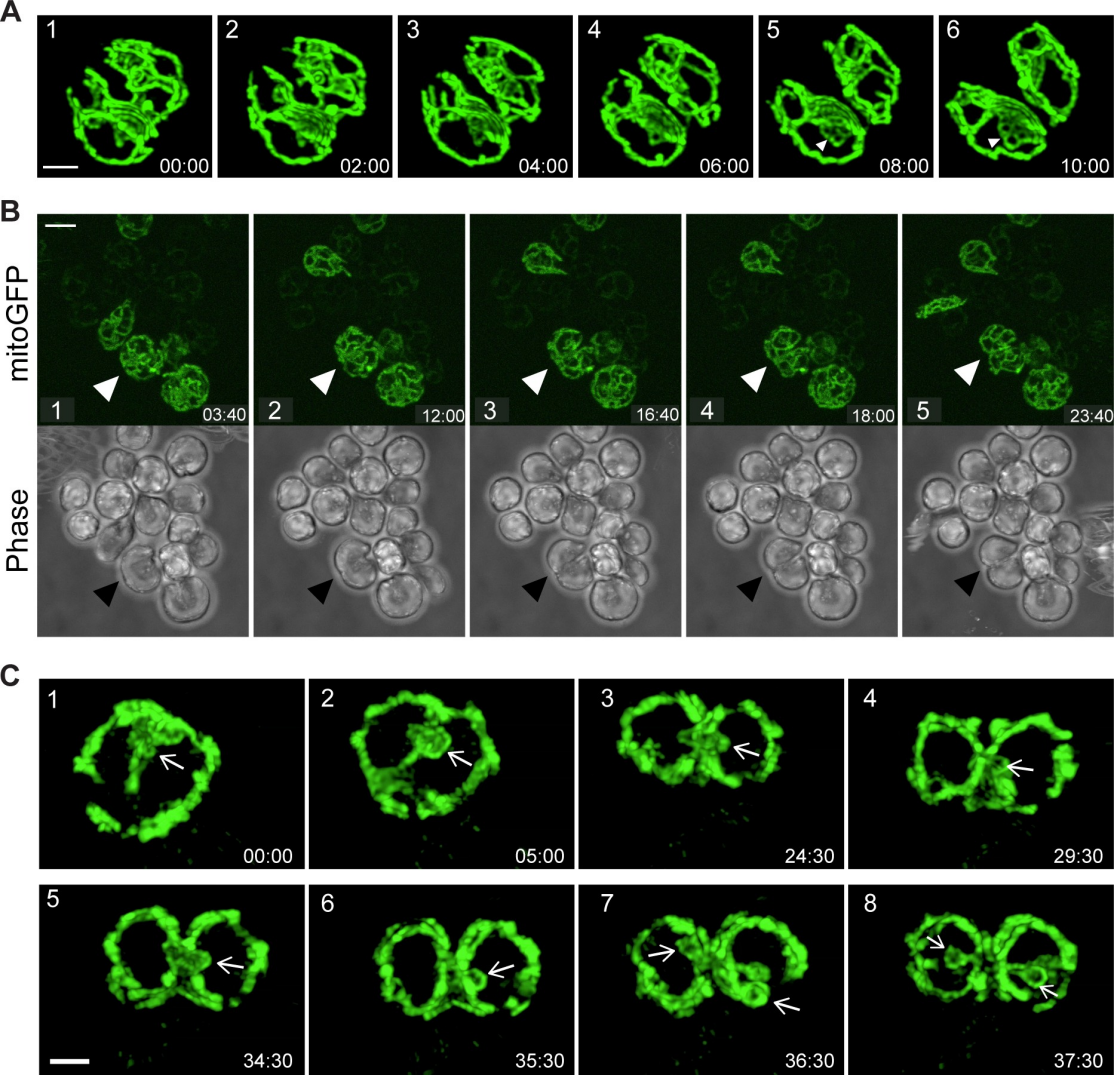
Mitochondrial division occurs just prior to cytokinesis in *C*. *fasciculata*. A) Time-lapse of mitochondrial/cell division in a swimming nectomonad immobilized in low melting point agarose. Image is a max projection of a Z-stack generated on a spinning disk confocal microscope and subjected to deconvolution. Times are given in mm:ss and numbers indicate the sequence of frames. The anterior of the cell is on the left side of each image. Complete mitochondrial division seems to occur between frames 3 and 4. A fenestrated sheet appears in the lower cell in frames 5 and 6 (arrowheads). Not all frames are shown (see S5 Movie). Scale bar is 2 μm. B) Mitochondrial and cell division of adherent haptomonads. Arrowheads indicate a dividing cell. Times are given in mm:ss and numbers indicate the sequence of frames. Not all frames are shown (see S6 Movie). Scale bar is 10 μm. C) Time lapse of a dividing haptomonad. The presumed position of the kinetoplast (identified by the small loop of mitochondrial matrix surrounding it, is indicated by white arrows. Numbers indicate the sequence of frames. Not all frames are shown (see S8 Movie). kDNA division occurs in frame 6–7. Scale bar is 2 μm.

Our fixed cell studies indicate a tight coupling between mitochondrial division and cytokinesis. To explore this relationship further, we visualized these processes in live cells. We observed that mitochondrial division occurs shortly before cytokinesis in both nectomonad and haptomonad cells (Fig 6A and 6B and S5–S7 Movies). The progress of mitochondrial division matched that of the cleavage furrow, beginning at the anterior end of the cell (between the two daughter flagellar pockets) and progressing posteriorly. The rest of the mitochondrion continued to undergo remodeling during the 2N1K and 2N2K stages, indicating that mitochondrial dynamics is not limited to a particular cell cycle stage (S7 Movie). However, we noted increased fusion of tubules at the midline of the cell at the position where the cytokinesis plane would pass through.

Our live cell imaging studies indicate that division of the kDNA is also temporally linked to cytokinesis. Prior to the initiation of cytokinesis, the kDNA moves from the anterior end to the center of the cell, straddling the division plane (Fig 6C and S8 and S9 Movies). The kDNA pocket appears as a single dark spot surrounded by a mitochondrial loop. This structure is larger than that in G1 cells since the kDNA has already replicated by this point. Shortly after the appearance of the cytokinesis furrow, the kDNA is divided in half and two smaller loops are visible (Fig 6C and S8 and S9 Movies). The process of kDNA division is rapid, occurring in under 10 minutes. To our knowledge, this is the first time this process has been observed in live cells.

## Discussion

Here we present evidence for rapid mitochondrial dynamics in the monoxenous kinetoplastid parasite, *C*. *fasciculata*. This remodeling occurs in both motile and non-motile cells and in all stages of the cell cycle. The morphogenic events of the *C*. *fasciculata* cell cycle, including changes in cell shape and the transient asymmetry of post-mitotic nuclei, resemble those in *Leishmania* [44]. Our data suggest that the majority of both cell growth and mitochondrial biogenesis occur in G1. Following nuclear mitosis, the mitochondrial network becomes more fused in the center before being divided in half in a process that is coincident with cytokinesis. Finally, our examination of live cells revealed that the replicated kDNA moves to the center of the cell following nuclear mitosis and divides shortly after the initiation of cytokinesis.

Our findings are consistent with a detailed study of mitochondrial structure in *T*. *brucei* bloodstream form (BSF) cells [47]. Typically, the BSF mitochondrion is a relatively uncomplicated tubular structure. However, by examining cells at different stages of the cell cycle, the authors present evidence for mitochondrial fission, branching and fusion. In addition, the mitochondrion in BSF *T*. *brucei* becomes highly fenestrated just prior to division and is then pruned back to a single tubule during cytokinesis. These mitochondrial remodeling events may be analogous to what we observe in dividing *C*. *fasciculata* cells, including increased midline fusion and conversion to closed networks. In both cases, changes in mitochondrial shape may allow even partitioning of the organelle into daughter cells. As in *C*. *fasciculata*, mitochondrial tubules in *T*. *brucei* were thinner during periods of rapid network growth. In fact, the broader range of mitochondrial sizes in 1N1K *C*. *fasciculata* cells may indicate that the mitochondrion actually overshoots its target size during this growth phase before returning to a steady-state during or just after nuclear mitosis. While mitochondria in BSF *T*. *brucei* and *C*. *fasciculata* differ in structure and function, dynamic remodeling in both species is coordinated with the cell cycle and is probably essential for maintenance of organelle structure during its expansion and division. Further, genetic conservation among kinetoplastids means that mitochondrial dynamics in these species is probably mediated by similar proteins with shared mechanisms for regulation.

Mitochondrial fusion/fission dynamics seem unlikely to operate in mitochondrial quality control in an organism with a single organelle and a single mitochondrial nucleoid, since any portion of the mitochondrion that separated from the network might lack mitochondrial DNA. Instead, we propose that mitochondrial dynamics in kinetoplastids may fulfill specific requirements related to 1) having a single mitochondrion and 2) having all mitochondrial genomes collected at one end of the organelle. In other eukaryotes, mitochondrial genomes are found throughout each mitochondrion. In kinetoplastids, all copies of the mitochondrial genome are topologically interlocked and found in a single structure called the kDNA [48]. The kDNA is physically attached to the basal body via a transmembrane system of filaments called the tripartite attachment complex [49], which retains it within the kDNA pocket region [47]. Fusion of distant parts of the network may accelerate the transport of lipids, proteins, mitochondrial transcripts, and other components beyond diffusion rates. This homogenization of the mitochondrial compartment would ensure that each daughter cell received a fully functional organelle upon cell division.

Mitochondrial dynamics in these organisms may have other functions as well. As parasites, most kinetoplastids can alter their metabolism to changing nutrient availabilities. Mitochondrial remodeling is a well-studied part of metabolic adaptation during the life cycle of *T*. *brucei*, during which mitochondrial shape and activity change dramatically. Long slender BSF cells are highly glycolytic and have an unbranched mitochondrion, while developmental stages in the tse-tse fly, including procyclic form, rely on mitochondrial energy production and have a highly-branched mitochondrion [50–52]. *C*. *fasciculata* has a single host and is most likely taken up by mosquitoes from a variety of habitats which could include pools of water and the surface of fruits or other plants. To survive, *C*. *fasciculata* parasites may also have to adjust their metabolism to make the most of their available nutrients. While we did not observe dramatic differences in mitochondrial structure or dynamics between nectomonads, which are more prevalent in the environment, and haptomonads, which are the primary form found in the mosquito [53], it should be noted that we generated these forms in culture in nearly identical nutrient environments. Whether mitochondrial shape and metabolism in *C*. *fasciculata* change in response to environmental conditions is an important question that could shed light on the conservation and possible functions of mitochondrial dynamics in a variety of parasites.

Although a few studies suggested the presence of mitochondrial fusion/fission dynamics [47, 54, 55], the single mitochondrion of kinetoplastid parasites had been presumed by some to be a relatively static network, with significant remodeling limited to division of the organelle prior to cell division [30] and differentiation between life cycle stages (in the case of *T*. *brucei* [50, 51]). One of the main justifications for this assumption has been a lack of clear orthologs for most mitochondrial dynamics proteins. In all kinetoplastids, mitochondrial fission is required for division of one organelle into two prior to or during cytokinesis. In *T*. *brucei*, this activity is mediated by TbDLP, which also plays a role in endocytosis [30]. There have been several studies on the two nearly identical TbDLPs [28–30]. Surprisingly, none of them show a clear effect on mitochondrial shape or dynamics such as that produced by depletion of the orthologous protein in yeast or mammalian cells [28–30]. The fact that knockdown of the only proposed mitochondrial fission activity in *T*. *brucei* has little effect on basic network structure implies that mitochondrial shape is maintained by other mechanisms. Without a “typical” role for DLP, kinetoplastids may serve as an excellent platform to discover dynamin-independent mechanisms of mitochondrial remodeling, which may be conserved in other organisms. For instance, a recent report on the dynamics of mitosomes in *Giardia intestinalis* parasites found that they undergo fission but not fusion, that fission is coordinated with the cell cycle, and that it occurs independently of dynamin-like proteins [56].

In further support of the existence of mitochondrial fusion in kinetoplastids, a mitofusin-like protein, TbMFNL, has been described [57]. Knockdown of this protein results in a highly fenestrated network in *T*. *brucei*, perhaps indicating that excess branches would typically be resolved through fusion. The presence of fusion implies that there must be an opposing process, and that the balance between these two activities maintains mitochondrial shape. In this way, the role of mitochondrial dynamics in kinetoplastids may resemble that in other eukaryotes.

Branching may be important for establishing mitochondrial network topology and may be mechanistically distinct from DLP-mediated fission. As mentioned above, analysis of mitochondrial structure during the cell cycle of *T*. *brucei* BSF cells provides evidence for dynamic branching and fusion of the mitochondrial network [47]. Consistent with our findings in *C*. *fasciculata*, fenestrated sheets were observed in BSF *T*. *brucei* and are thought to resolve into tubules [55]. Whether these events are mediated by proteins that are conserved in the eukaryotic lineage, or whether they involve novel proteins specific to kinetoplastids, remains to be discovered. In either case, branching activities may need to be held in balance with periodic membrane fusion events in order to preserve the shape of the mitochondrial network.

By imaging live cells, we were able to observe mitochondrial division and, indirectly, kDNA division. Division of the mitochondrion in *C*. *fasciculata* is closely coordinated with cytokinesis, as has been observed for *T*. *brucei* [30, 47, 55]. However, the mechanism of kDNA division is different between these two species [58]. In *C*. *fasciculata*, *Leishmania*, *and T*. *cruzi*, the kDNA divides in a single step mediated by an unknown enzymatic activity. By visualizing the kinetics of the loop of mitochondrion that contains the kDNA, we were able to image cells at the moment in which one kDNA network became two, shortly after the appearance of the cleavage furrow. Division occurred rapidly (under 10 minutes), making it difficult to imagine that the precise cutting and resealing of the thousands of DNA circles in this complex structure took place in this time frame. Possibly the two daughter kDNAs have already been divided at an earlier stage but remain tethered until the final division event. Integration of the precise timing of kDNA division into our understanding of the mitochondrial duplication and division cycle provides a more complete picture of the highly-orchestrated cell cycle by which these organisms maintain their single-copy organelles.

Under certain conditions, yeast and mammalian mitochondria form an interconnected network [59], meaning that this mitochondrial configuration is not unique to kinetoplastids. If mitochondrial dynamics is broadly conserved throughout eukaryotic evolution, study of this process in early-diverging eukaryotes such as kinetoplastids could provide important insights into its evolution and essential functions, while possibly explaining the metabolic adaptability of these parasites to different environmental conditions. Further, it is possible that intra-organelle fusion and fission reactions in *C*. *fasciculata* are mediated by novel or highly-diverged proteins, the study of which could reveal important mechanisms that will be applicable to the study of other eukaryotic model systems.

## Supporting information

S1 Fig Clonal mitoGFP-expressing *C. fasciculata* lines were screened for GFP expression. A) Protein lysates from non-clonal (populations) and clonal lines were screened by western blot with anti-GFP antibody. Arrow indicates mitoGFP band. B) An identical gel as that used to generate the blot in A was run in parallel and stained with Coomassie as a loading control.
(TIF)

S2 Fig Quantitation of different mitochondrial morphologies in *C. fasciculata*. A) Representative images of mitochondrial networks consisting of mainly thin tubules, a mixture of thin and thick tubules, and mainly thick tubules. B) Quantitation of different types of mitochondrial networks in cells at different stages of the cell cycle. C) Distribution of thick tubules in different areas of the cell according to cell cycle stage.
(TIF)

S1 Movie The mitochondrion is dynamic in *C. fasciculata*. A representative swimming *C*. *fasciculata* cell expressing mitoGFP immobilized in agarose and imaged on a spinning disk confocal microscope. Maximum projections are shown including the frames shown in Fig 4A.
(MOV)

S2 Movie Mitochondrial dynamics in *C. fasciculata* nectomonads. Swimming *C*. *fasciculata* cells expressing mitoGFP were immobilized in agarose and imaged on a spinning disk confocal microscope. Maximum projections of a representative cell are shown including the frames shown in Fig 4A.
(MOV)

S3 Movie Mitochondrial dynamics in *C. fasciculata* haptomonads. Adherent *C*. *fasciculata* cells expressing mitoGFP were imaged on a laser scanning confocal microscope. Maximum projections of a representative rosette are shown and correspond to the frames shown in Fig 5A.
(MOV)

S4 Movie Dynamic fenestrated sheets appear in *C. fasciculata* mitochondria. An adherent *C*. *fasciculata* haptomonad expressing mitoGFP was imaged on a laser scanning confocal microscope. Maximum projections are shown which have been color coded according to depth and which correspond to frames shown in Fig 5B.
(MOV)

S5 Movie Coordination of mitochondrial division and cytokinesis in nectomonads. Maximum projection (deconvolved) of a swimming nectomonad *C*. *fasciculata* cell undergoing cytokinesis. The mitochondrion was imaged using mitoGFP. Time-lapse corresponds to frames shown in Fig 6A.
(MOV)

S6 Movie Coordination of mitochondrial division and cytokinesis in haptomonads. A rosette of adherent *C*. *fasciculata* cells expressing mitoGFP. The cell at the bottom left is undergoing cytokinesis. Cleavage furrow ingression begins at 01:20 (mm:ss). Time-lapse of maximum projections corresponds to frames shown in Fig 6B.
(MOV)

S7 Movie Mitochondrial dynamics during cell division of haptomonads. Maximum projection of a rosette of adherent *C*. *fasciculata* cells expressing mitoGFP. The cell at the right is undergoing mitochondrial division/cytokinesis. The top and bottom slices of the deconvolved Z-stack were removed in order to clearly visualize the division events.
(MOV)

S8 Movie Live-cell imaging of kDNA division in *C. fasciculata*. Time-lapse of an adherent haptomonad *C*. *fasciculata* cell expressing mitoGFP. Several frames of the Z-stack have been removed from the maximum projections in order to clearly show the process of kDNA divison. Time-lapse corresponds to frames shown in Fig 6C.
(MOV)

S9 Movie The timing of kDNA division in *C. fasciculata*. Deconvolved maximum projection of a *C*. *fasciculata* rosette expressing mitoGFP. The upper middle cell is in the initial stages of cytokinesis. The cell is oriented such that the anterior of the cell (where cleavage furrow ingression begins) is facing down. Division of the kDNA can also be observed.
(MOV)
