# Supplementary figures and images for: The single mitochondrion of the kinetoplastid parasite Crithidia fasciculata is a dynamic network
Source: PLoS One. 2018 Dec 28;13(12):e0202711. doi: 10.1371/journal.pone.0202711 (PMC6310254; doi:10.1371/journal.pone.0202711)

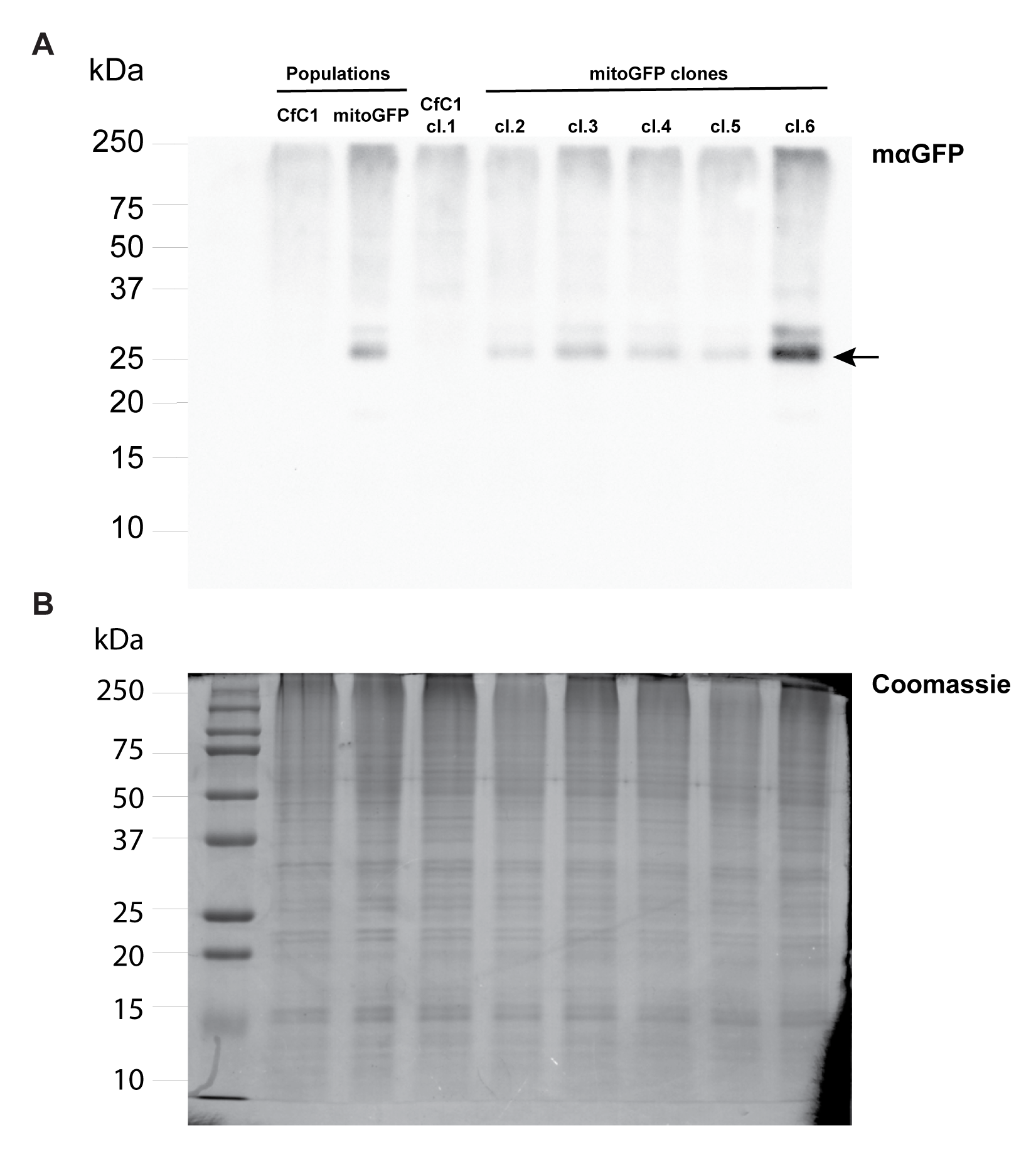

Supplement: S1 Fig — A) Protein lysates from non-clonal (populations) and clonal lines were screened by western blot with anti-GFP antibody. Arrow indicates mitoGFP band. B) An identical gel as that used to generate the blot in A was run in parallel and stained with Coomassie as a loading control. (TIF) [file pone.0202711.s001.tif]

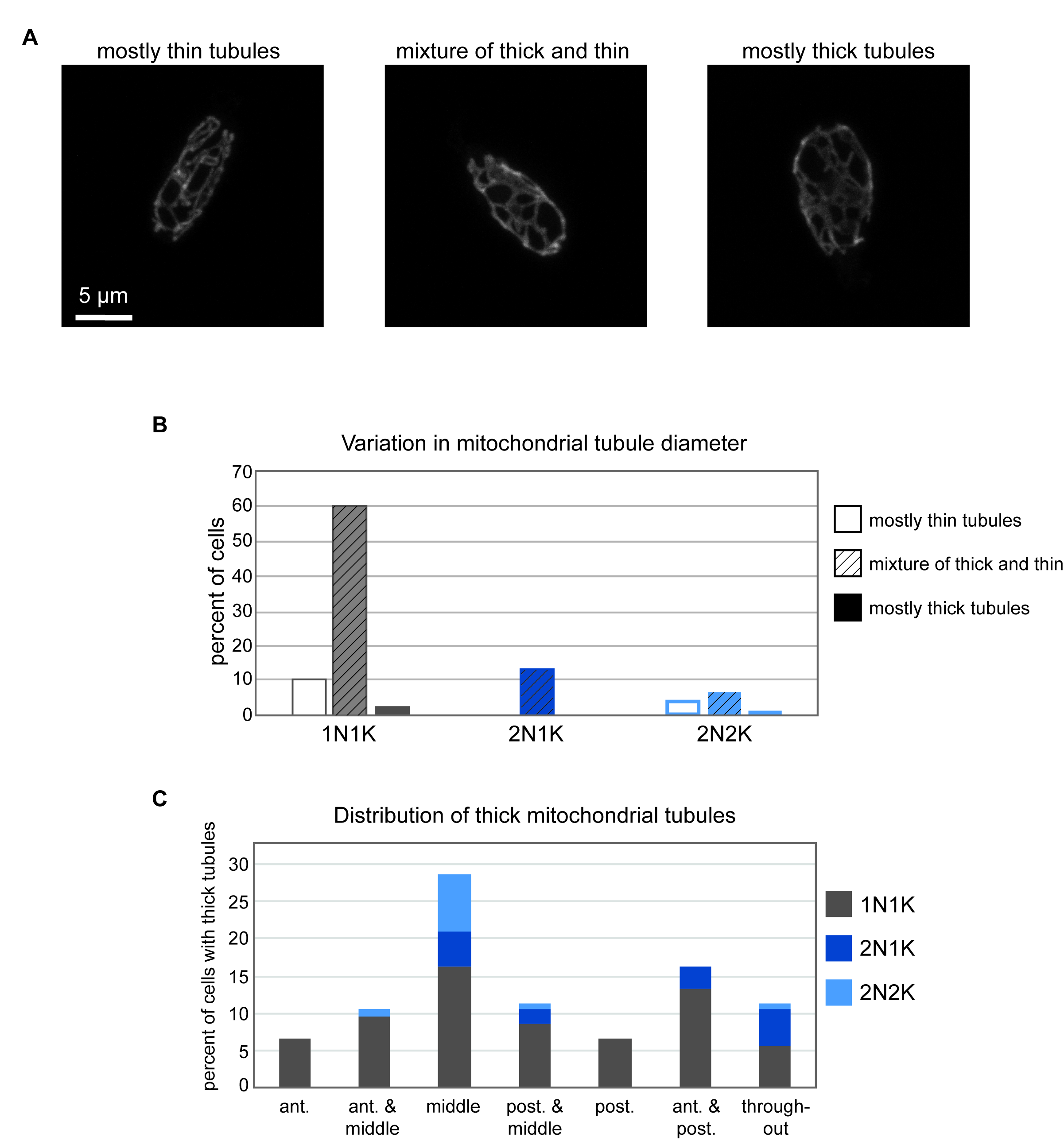

Supplement: S2 Fig — A) Representative images of mitochondrial networks consisting of mainly thin tubules, a mixture of thin and thick tubules, and mainly thick tubules. B) Quantitation of different types of mitochondrial networks in cells at different stages of the cell cycle. C) Distribution of thick tubules in different areas of the cell according to cell cycle stage. (TIF) [file pone.0202711.s002.tif]
